# Six-month follow up of a randomized clinical trial-phase I study in Indonesian adults and children: Safety and immunogenicity of *Salmonella typhi* polysaccharide-diphtheria toxoid (Vi-DT) conjugate vaccine

**DOI:** 10.1371/journal.pone.0211784

**Published:** 2019-02-13

**Authors:** Bernie Endyarni Medise, Soedjatmiko Soedjatmiko, Iris Rengganis, Hartono Gunardi, Rini Sekartini, Sukamto Koesno, Hindra Irawan Satari, Sri Rezeki Hadinegoro, Jae Seung Yang, Jean-Louis Excler, Sushant Sahastrabuddhe, Mita Puspita, Rini Mulia Sari, Novilia Sjafri Bachtiar

**Affiliations:** 1 Department of Child Health, Faculty of Medicine Universitas Indonesia, Dr. Cipto Mangunkusumo General National Hospital, Jakarta, Indonesia; 2 Department of Internal Medicine, Faculty of Medicine Universitas Indonesia, Dr. Cipto Mangunkusumo General National Hospital, Jakarta, Indonesia; 3 International Vaccine Institute, Seoul, Republic of Korea; 4 PT. Bio Farma, Bandung, Indonesia; Public Health England, UNITED KINGDOM

## Abstract

**Introduction:**

There is a high global incidence of typhoid fever, with an annual mortality rate of 200,000 deaths. Typhoid fever also affects younger children, particularly in resource-limited settings in endemic countries. Typhoid vaccination is an important prevention tool against typhoid fever. However, the available polysaccharide typhoid vaccines are not recommended for children under 2 years of age. A new typhoid conjugate Vi-diphtheria toxoid (Vi-DT) vaccine has been developed for infant immunization. We aimed to define the safety and immunogenicity of the Vi-DT vaccine among adults and children in Indonesia.

**Methods:**

An observational, blinded, comparative, randomized, phase I safety study in two age de-escalating cohorts was conducted in East Jakarta, Indonesia, from April 2017 to February 2018. We enrolled 100 healthy subjects in 2 age groups: adults and children (18–40 and 2–5 years old). These groups were randomized into study groups (Vi-DT vaccine), and comparator groups (Vi-polysaccharide (Vi-PS) vaccine and another additional vaccine) which was administered in 4 weeks apart. Subjects were followed up to six months.

**Result:**

One hundred healthy adults and children subjects completed the study. The Vi-DT and Vi-PS vaccines showed no difference in terms of intensity of any immediate local and systemic events within 30 minutes post-vaccination. Overall, pain was the most common local reaction, and muscle pain was the most common systemic reaction in the first 72 hours. No serious adverse events were deemed related to vaccine administration. The first and second doses of the Vi-DT vaccine induced seroconversion and higher geometric mean titers (GMT) in all subjects compared to that of baseline. However, in terms of GMT, the second dose of Vi-DT did not induce a booster response.

**Conclusion:**

The Vi-DT vaccine is safe and immunogenic in adults and children older than two years. A single dose of the vaccine is able to produce seroconversion and high GMT in all individuals.

## Introduction

Typhoid fever remains a serious systemic infection and a public health threat throughout the world, particularly in resource-limited settings and countries, including some parts of Indonesia, which lack of clean drinking water, hygiene, and good sanitation. This enteric disease is caused by *Salmonella enterica* serovar typhi and spreads through the fecal-oral route.[1–4] Although mostly endemic, *S*. *typhi* has epidemic potential and causes 60% to 80% of typhoid infections in humans.[1,4–6]

The global burden of typhoid fever is estimated at 26.9 million cases, with an annual mortality rate of 200,000 deaths. School-aged children (5–15 years old) are disproportionately affected. In some endemic areas, children under 5 years old have incidence rates similar to or exceeding those of school-aged children.[7–15] Other studies suggest that global mortality rates can reach 4%, with 90% of deaths occurring in developing countries in Asia. Although the rates have currently decreased, additional data suggest a similar burden in Sub-Saharan Africa.[7,15,16] The incidence of typhoid fever ranges globally from 15.3 per 100,000 people aged 5–60 years in China to 451.7 per 100,000 children aged 2–15 years in Pakistan.[1,15] A study in an urban area in Kenya showed that the overall crude incidence of bacteremia caused by *S*. *typhi* was 247 cases per 100,000 person-years of observation (pyo) with the highest rates in children 5–9 years old (596 per 100,000 pyo) and 2–4 years old (521 per 100,000 pyo); rural areas showed rates of 29 cases per 100,000 pyo with low rates in children 2–4 and 5–9 years old (28 and 18 cases per 100,000 pyo, respectively). The adjusted incidence rates were the highest in 2–4 years old urban children (2,243 per 100,000 pyo); these rates were >15-fold higher than the rates in rural children of the same age group.[17] A study in Malawi, East Africa during 1998–2004 reported 4,956 cases of invasive non-typhoidal Salmonellosis (75% *S*. *typhimurium*, 21% *S*. *enteritidis*) from 62,778 blood samples.[18]

In a cohort study conducted in 5,570 children in Pakistan, 16 children showed a positive blood culture of *S*. *typhi*, including 7 cases (43.7%) in children younger than 2 years old and 4 cases in children younger than 12 months of age.[12] In a multicenter study conducted in five Asian countries (Diseases of the Most Impoverished Program of the International Vaccine Institute), the incidence of typhoid fever in Indonesia was 148.7 per 100,000 people per year for children aged 2–4 years old, 180.3 for children aged 5–15 years old, and 51.2 for those >16 years old, with the average onset occurring at the age of 10.2 years.[15] In a study conducted in Jatinegara, East Jakarta, Indonesia, 1,019 cases of fever were reported in those 3 to 59 years old with 88 cases (9%) of *S*. *typhi* and 26 cases (3%) of *S*. *paratyphi A* identified.[19]

Because of the limited availability of blood culture services and surveillance techniques to measure the disease incidence, the disease burden of typhoid fever remains difficult to assess in developing and endemic countries. The understanding of the magnitude of complications and deaths in the population is also limited.[8] With the recent increase in antimicrobial resistance, the treatment for typhoid has become expensive and prolonged.[2,8] Although most of developing countries attempt to reduce the typhoid burden by improving sanitation, hygiene and infrastructure, it remains a challenging goal with long-term implications. Hence, vaccination against typhoid serves as an effective prevention measure in the short-to-medium term, particularly when combined with other prevention efforts.[20–23]

There is increasing awareness about the burden of typhoid in children under 2 years of age, particularly in endemic countries.[8,12,22] However, the currently available typhoid vaccines, parenteral unconjugated Vi polysaccharide (Vi-PS) and live oral Ty21a, are not recommended for use in infants and children under 2 years of age. Therefore, it is important to develop a typhoid vaccine that can provide long-term immunity, particularly for children under 2 years old who live in endemic countries.[8,20–22] Regarding the other polysaccharide vaccines, Vi-PS generates a T-cell independent immune response and hence is not effective in children under 2 years of age. For a better immune response, we need to conjugate a carrier protein to the polysaccharide antigen and cause it to shift from T-independent to T-dependent immunity. The strategy of conjugating the polysaccharide to a carrier protein to overcome this limitation has been successfully used for pneumococcal, meningococcal and Hib polysaccharide vaccines.[20–23] Some studies have reported results of typhoid Vi-PS vaccines which were conjugated with various carrier proteins.[20–24]

The International Vaccine Institute (IVI), Seoul, Republic of Korea, has developed a typhoid conjugate vaccine (TCV) candidate, which Vi-PS is conjugated to the diphtheria toxoid (DT) carrier protein, namely “Vi-DT”. Vi-PS is harvested from *S*. *typhi* strain C6524, which is a clinical isolate from India. The conjugation chemistry involves first binding adipic acid dihydrazide (ADH) spacer molecules to the DT carrier protein, followed by binding this modified DT to Vi-PS. The process was optimized to yield consistent results. In animal experiments, Vi-DT was found to be far more immunogenic than Vi-PS alone.[24]

The phase I study of this new vaccine were conducted in two countries, Philippine and Indonesia. Study in Filipino adults and children, which was done a year earlier than in Indonesia, showed a promising safety result in the two-month follow up.[25]

In Indonesia, after receiving this technology from IVI, PT Bio Farma adopted the process at their facilities and conducted the preclinical toxicology studies. The results were encouraging, and a first-in-human study was conducted in Jakarta, with a longer follow-up period than in Philippine. We report the Vi-DT vaccine’s safety and immunogenicity results from a phase I study among Indonesian adults and children for a six-months follow up.

## Methods

### Ethics statement

The study protocol and all amendments were reviewed and approved by the Health Research Ethics Committee of the Faculty of Medicine, Universitas Indonesia (1015/UN2.F1/ETIK/2016) in compliance with local law (23/UN2.F1/ETIK/I/2017). This study has also been registered in ClinicalTrials.gov ID: NCT03109600. Prior to the study, informed consent was obtained from all subjects, including adult subjects (18–40 years old) and the parents or guardians of the child subjects (2–5 years old).

### Study design

We conducted an observer-blinded, comparative, randomized, phase I safety and immunogenicity study in two age de-escalating cohorts. Generally, in phase I clinical studies, initial testing of a vaccine is conducted in only small numbers (e.g., 25) of healthy adults. We enrolled 100 healthy subjects in two age groups: 18 to 40 years old (adults) and 2 to 5 years old (children). Age de-escalation grouping was used to enroll younger subjects in the absence of any safety concerns in adult subjects. The two age groups were then divided into 4 arms as shown in Table 1.

**Table 1:** Study arms and dosing schemes.

Age group	Group	n	Number of vaccine doses	Volume injected
18–40 years	I	25	2 doses of Vi-DT (4-week interval)	0.5 mL
	II	25	1 dose of Vi-PS 1 dose of the influenza HA vaccine	0.5 mL
2–5 years	III	25	2 doses of Vi-DT (4-week interval)	0.5 mL
	IV	25	1 dose of Vi-PS 1 dose of the 13-valent pneumococcal polysaccharide conjugated vaccine	0.5 mL

### Study area and time

This study was conducted in the Jatinegara District Primary Health Center (PHC) area, East Jakarta, Indonesia. The subjects were recruited by the research team from the Department of Child Health, in collaboration with the Department of Internal Medicine, Faculty of Medicine Universitas Indonesia, Dr. Cipto Mangunkusumo General National Hospital. Recruitment for the adult group lasted from April 17^th^ to May 17^th^, 2017; follow-up was conducted until November 29^th^, 2017. The enrolled subjects underwent 4 initial clinical visits where initial data was obtained and vaccinations were administered; they continued to be monitored for a six-month period. Recruitment of the children lasted from July 17^th^, 2017 (a month after second vaccination in adult group) to August 1^st^, 2017. Their follow-up was conducted until February 19^th^, 2018.

### Procedures

Subjects who involved in another trial, had fever (axillary temperature >37.5°C), had a known history of allergy to any component of the vaccines, had a history of uncontrolled coagulopathy or blood disorders contraindicating intramuscular injection, had in the previous 4 weeks received a treatment likely to alter the immune response (intravenous immunoglobulins, blood-derived products or corticotherapy for more than 2 weeks), had any abnormality or chronic disease that might interfere with the safety of the subject and compromise the trial objectives, had previously received any vaccines against typhoid fever or been immunized with any vaccine within the prior 4 weeks, and/or were expecting to receive other vaccines within 60 days following the first dose, were excluded. Other exclusion criteria were those who had previously ascertained or suspected typhoid fever within 3 months prior to immunization, those planning to move from the study area before the end of the study period, and adults who had a history of alcohol or substance abuse or were pregnant or lactating.

Initial data were collected at V0 (three days prior to the study). All adult subjects underwent blood laboratory tests (biochemistry and hematology), a urine test, chest X-ray, electrocardiography (ECG), and physical examination performed by an internist. The adult subjects who met the inclusion criteria were allocated a study-specific inclusion number from 001 to 050, and they were randomly assigned into two groups out of the four-block randomization for each age group. The investigators allocated each subject based on their inclusion number and randomization code. The recruitment process was conducted by the age de-escalating method, starting with eight subjects in the adult group. Safety during the first 3 days and the laboratory results on the 7^th^ day post-first vaccination was assessed and reviewed. The safety review of the first eight subjects showed good results, the study then continued with the recruitment of 12, 14, and 16 subjects with the same procedures. After all the subjects completed V3 in the adult group, an interim safety review was conducted before recruiting the children group with same steps of de-escalation. Children only underwent a physical examination prior to the study. The children were then allocated an inclusion number from 051 to 100 and randomly assigned to two groups of the four-block randomization. Blood sample was taken prior vaccination.

We compared Vi-DT with Vi-PS in the adult and children subjects. The study group received Vi-DT and the comparator group received Vi-PS vaccine at visit 1 (V1). In the adult subjects, seven days after first vaccination, we obtained another blood sample. At V2, 4 week-interval (-4/+7days), after another blood sample was obtained in both groups, we administered the second Vi-DT vaccine in the study group, and we gave the influenza HA vaccine (Flubio, Bio Farma) to adult comparator group and the 13-valent pneumococcal PS conjugate vaccine (Prevenar, Wyeth) to children comparator group as the second dose. These vaccines have different packaging features. Therefore, an unblinded team (arm pediatricians and investigational product officer) was appointed to administer the vaccines.

### Blood samples

All blood samples were processed to obtain sera and were stored between −20°C to −80°C. Four milliliters of blood was necessary for the immunogenicity assessment.

### Investigational vaccine

Each dose of the Vi-DT vaccine contains a purified Vi capsular polysaccharide of *S*. *typhi* (25 ug), 2-phenoxyethanol (preservative) (5 mg), and phosphate buffer solution q.s. in a total volume of 0.5 mL. The Vi-DT vaccine was injected intramuscularly in the left deltoid region.

### Adverse events following immunization (AEFI)

All subjects were provided with an observation diary card to assess and record information for local/systemic reactions for 28 days following immunization, with special attention within the first three days. In the adult subjects, an additional blood sample was obtained on day 7 after the first vaccination to review any biological AEFI. Systemic reactions were recorded in the observation card up to 6 months after the last vaccination. We recorded any local and systemic, immediate and delayed, and serious adverse events (SAEs). Reports for every outcome of vaccination were routinely reviewed by the Data Safety Monitoring Board (DSMB).

### Immunogenicity

Anti-Vi antibodies were assessed by a Vi-IgG ELISA at the Immunology Laboratory of the Clinical Trial Department of Bio Farma. This method had been transferred by IVI, validated by the Clinical Trial Department of Bio Farma and approved by the Quality Assurance Division. Bio Farma used in-house reference serum that has been optimized using Vi IgG_R1, 2011_ as a reference; anti-Vi-IgG levels were expressed in μg/mL. The seroconversion rates after the first and second doses and the geometric mean titers (GMT) with their 95% CIs were described at V0, V2 and V3. Because there is not yet a correlate of protection established for typhoid, a four-fold increase from the baseline value was considered the measure of seroconversion.

### Data analysis and statistics

All data were analyzed using SPSS 20. Vaccine safety was analyzed by computing the number and percentage of any adverse events experienced by subjects. Seroconversion and GMT will be presented in crude rates with their 95% confidence intervals (computed using the exact binomial probability).

## Results

### Study population

**Fig 1:**
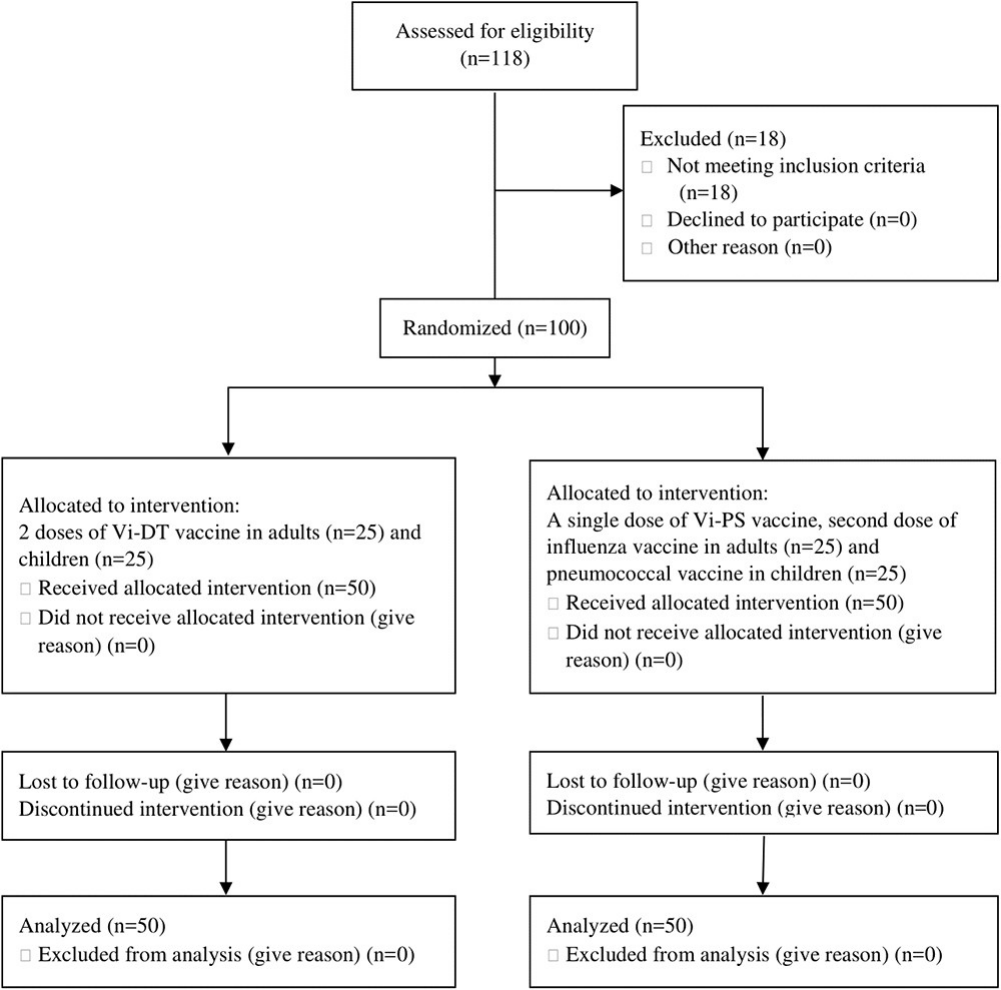
Consort diagram.

Out of 118 eligible subjects, we enrolled a total of 100 healthy subjects, including 50 adults and 50 children. We randomized them into study (Vi-DT vaccine) and comparator (Vi-PS vaccine) group, each consisted of 25 adults and 25 children. All subjects completed the study (Fig 1). All adult subjects were clinically healthy prior to vaccination as shown by the results of the physical examination, chest X-ray, urinalysis and blood tests. Hematological and biochemical values were within normal limits, and there was no significant deviation on day 7 after the first immunization in adult subjects. All children were also clinically healthy prior to vaccination based on physical examination by a pediatrician. Demographic and baseline characteristics of the subjects are presented in Table 2. In adult group, most of subjects had high grade of education, meanwhile most of parents in children group had middle education level.

**Table 2:** Demographic and baseline characteristics of the subjects.

Description	Adults		Children	
	Control n = 25	Vi-DT n = 25	Control n = 25	Vi-DT n = 25
**Sex, n**				
Female	14	12	9	12
Male	11	13	16	13
**Own occupation, n**				
Adult/father’s subject	19	22	25	23

### Safety evaluation

A proportion of any immediate and delayed adverse events after the first and second vaccinations was shown in Table 3. Only proportion of systemic event at 31 minutes to 24 hours after the first vaccination in adult group revealed a significant difference (p<0.05) between study and control group. In terms of duration and intensity, both vaccines have similar safety outcomes with majority in mild level (Tables 4 and 5).

**Table 3:** Immediate and delayed adverse events after the first and second vaccinations.

Description	First vaccination												Second vaccination											
	Adults					Children					All		Adults					Children					All	
	Control		Vi-DT		p	Control		Vi-DT		p			Control		Vi-DT		P	Control		Vi-DT		p		
	n	%	n	%		n	%	n	%		n	%	n	%	n	%		n	%	n	%		n	%
Any immediate reaction																								
• any immediate local event	5	20	3	12	0.702^b^	3	12	3	12	1.000^b^	14	14	4	16	4	16	1.000^b^	0	0	1	4	1.000^b^	9	9
• any immediate systemic event	3	12	1	4	0.609^b^	0	0	0	0	n/a	4	4	1	4	1	4	1.000^b^	0	0	1	4	1.000^b^	3	3
Any delayed adverse event 31 min-24 hours																								
• any delayed local reaction	9	36	7	28	0.544^a^	9	36	10	40	0.771^a^	35	35	6	24	4	16	0.480^b^	2	8	5	20	0.417^b^	14	14
• any delayed systemic event	13	52	6	24	0.041^a^	7	28	7	28	1.000^a^	33	33	3	12	6	24	0.463^b^	0	0	5	20	0.050^b^	14	14
Any delayed adverse event 24 hours-48 hours																								
• any delayed local reaction	4	16	3	12	1.000^b^	3	12	1	4	0.609^b^	11	11	0	0	0	0	n/a	0	0	0	0	n/a	0	0
• any delayed systemic event	4	16	1	4	0.349^b^	1	4	1	4	1.000^b^	7	7	0	0	0	0	n/a	0	0	0	0	n/a	0	0
Any delayed adverse event 48 hours-72 hours																								
• any delayed local reaction	0	0	0	0	n/a	0	0	0	0	n/a	0	0	0	0	1	4	1.000^b^	0	0	0	0	n/a	1	1
• any delayed systemic event	3	12	2	8	1.000^b^	1	4	1	4	1.000^b^	7	7	0	0	0	0	n/a	0	0	0	0	n/a	0	0
Any delayed adverse event 72 hours-7 days																								
• any delayed local reaction	0	0	0	0	n/a	0	0	0	0	n/a	0	0	n/a	n/a	n/a	n/a	n/a	n/a	n/a	n/a	n/a	n/a	n/a	n/a
• any delayed systemic reaction	1	4	1	4	1.000^b^	1	4	1	4	1.000^b^	4	4	n/a	n/a	n/a	n/a	n/a	n/a	n/a	n/a	n/a	n/a	n/a	n/a
Any delayed adverse event 8 days-28 days																								
• any delayed local reaction	0	0	0	0	n/a	0	0	0	0	n/a	0	0	n/a	n/a	n/a	n/a	n/a	n/a	n/a	n/a	n/a	n/a	n/a	n/a
• any delayed systemic event	0	0	0	0	n/a	6	24	5	20	0.733^a^	11	11	n/a	n/a	n/a	n/a	n/a	n/a	n/a	n/a	n/a	n/a	n/a	n/a
Any delayed adverse event 72 hours-28 days																								
• any delayed local reaction	n/a	n/a	n/a	n/a	n/a	n/a	n/a	n/a	n/a	n/a	n/a	n/a	0	0	0	0	n/a	0	0	0	0	n/a	0	0
• any delayed systemic event	n/a	n/a	n/a	n/a	n/a	n/a	n/a	n/a	n/a	n/a	n/a	n/a	2	8	2	8	1.000^b^	5	20	9	36	0.208^b^	18	18

^a^ *p* values were calculated using Chi square

^b^ *p* values were calculated using Fisher

**Table 4:** The intensity of any local and systemic events within 30 minutes post-vaccination.

Description	Intensity																
	Adults							Children							All		
	Control			Vi-DT			p	Control			Vi-DT			p			
	Mild	Moderate	Severe	Mild	Moderate	Severe		Mild	Moderate	Severe	Mild	Moderate	Severe		Mild	Moderate	Severe
**Local reaction**																	
Pain	5	1	1	4	3	0	1.000	3	0	0	4	0	0	0.687	16	4	1
Redness	2	0	0	0	0	0	0.153	0	0	0	0	0	0	n/a	2	0	0
Swelling	0	0	0	0	0	0	n/a	0	0	0	0	0	0	n/a	0	0	0
Induration	0	0	0	0	0	0	n/a	0	0	0	0	0	0	n/a	0	0	0
Other	0	0	0	0	0	0	n/a	0	0	0	0	0	0	n/a	0	0	0
**Systemic event**																	
Fever	0	0	0	0	0	0	n/a	0	0	0	1	0	0	1.000	1	0	0
Fatigue	2	0	0	0	0	0	0.153	0	0	0	0	0	0	n/a	2	0	0
Muscle pain	2	1	0	2	0	0	0.463	0	0	0	0	0	0	n/a	4	1	0
Other	0	1	0	0	0	0	1.000	0	0	0	0	0	0	n/a	0	1	0

Statistics test using chi-square Mantel-Haenszel test for trend

**Table 5:** Intensity of any local and systemic event (>30 minutes-72 hours) post-vaccination.

Description	Intensity																
	Adults							Children							All		
	Control			Vi-DT			p	Control			Vi-DT			p			
	Mild	Moderate	Severe	Mild	Moderate	Severe		Mild	Moderate	Severe	Mild	Moderate	Severe		Mild	Moderate	Severe
**Local reaction**																	
Pain	6	4	2	5	7	2	0.676	8	4	1	10	2	1	0.538	29	17	6
Redness	1	0	0	1	0	0	1.000	3	1	0	5	0	0	0.444	10	1	0
Swelling	2	2	0	1	1	0	0.417	4	1	0	3	0	0	0.332	10	4	0
Induration	3	2	0	0	1	0	0.176	3	1	0	2	0	0	0.294	8	4	0
Other	5	0	0	1	0	0	0.189	0	0	0	0	0	0	n/a	6	0	0
**Systemic event**																	
Fever	0	0	0	1	0	1	0.203	2	0	0	4	1	1	0.410	7	1	2
Fatigue	6	1	0	3	4	0	0.525	2	1	1	5	1	0	1.000	16	7	1
Muscle pain	11	3	1	4	5	0	0.300	4	1	1	6	2	0	0.839	25	11	2
Other	3	1	0	5	1	0	1.000	1	0	0	1	0	0	1.000	10	2	0

Statistics test using chi-square Mantel-Haenszel test for trend

Muscle pain with mild intensity was the most common systemic reaction and found higher in adult control group. There were six SAEs reported in our study that occurred in four adult subjects and one child subject. One adult was diagnosed with dengue hemorrhagic fever, one with orchitis caused by parotitis, one with fever and one with cholelithiasis. One child subject experienced upper respiratory infection twice in two different months within the six-month follow up period. None of the SAEs was related to vaccination or the study procedures. No other adverse event was reported in the six-month follow up period.

### Immunogenicity evaluation

All blinded blood samples at V0, V2 and V3 were tested between November 10 and 22, 2017. The randomization codes were unblinded on November 28, 2017. All (100%) adults and children subjects who received Vi-DT showed a >4-fold increase in anti-Vi-IgG antibodies within 28 days after the first and second immunizations compared to baseline values (Table 6).

**Table 6:** The percentage of subjects with an increase in anti-Vi-IgG antibody titers >4 times, 28 days after the first and second immunizations compared to baseline.

Group	Increment of antibody titers V0 to V2		Increment of antibody titers V0 to V3	
	<4 times n (%)	≥4 times n (%)	<4 times n (%)	≥4 times n (%)
**Adult**				
Control	3 (12)	22 (88)	2 (8)	23 (92)
Vi-DT	0 (0)	25 (100)	0 (0)	25 (100)
**Children**				
Control	3 (12)	22 (88)	4 (16)	21 (84)
Vi-DT	0 (0)	25 (100)	0 (0)	25 (100)

The first dose of Vi-DT vaccination both in adult and child groups produced a significant increase of GMT compared to Vi-PS (p<0.001). However, the second dose of Vi-DT did not induce a booster response (Table 7).

**Table 7:** Geometric mean titer (GMT) of anti-Vi-IgG antibodies following immunization.

Group	GMT V0 (95% CI)	GMT V2 (95% CI)	GMT V3 (95% CI)
	Pre-immunization	28 days post-1^st^ immunization	28 days post-2^nd^ immunization
**Adult**			
Control	0.011 (0.00–0.00)	12.696 (8.11–21.33)	14.846 (9.55–23.82)
Vi-DT	0.041 (0.00–0.00)	86.118 (64.49–189.16)	62.275 (52.20–96.27)
**Children**			
Control	0.001 (0.00–0.00)	10.221 (7.60–13.73)	12.591 (9.47–17.04)
Vi-DT	0.008 (0.00–0.00)	95.823 (80.33–115.36)	79.897 (67.21–95.85)

## Discussion

Although typhoid vaccination is not yet a part of Indonesia’s Expanded Program for Immunization (EPI), the Indonesian Pediatric Society (IPS) immunization schedule still recommends all 2 years old children to receive the typhoid Vi-PS vaccine with booster doses every three years.[26,27] However, a study in two sub-districts of North Jakarta, Indonesia reported that the implementation of a large-scale, school-based typhoid Vi-PS vaccination campaign was logistically feasible, safe, and minimally disruptive to regular school activities, and had high rates of parental acceptance.[28]

Currently available Vi-PS vaccines can only be given to children aged 2 years or older. In endemic areas, *S*. *typhi* could be found in children younger than 2 years.[12] The available Vi-PS has some limitations due to T-cell independent properties which provide limited protection in infancy an toddlers. The development of conjugate vaccines overcome limitation associated with available polysaccharide vaccine. Conjugated vaccines have been proven to be effective against invasive bacterial diseases in neonates and young children.[29–30] Hence, studies of previous TCV have shown promising safety and immunogenicity in humans.[11,13,21,25,29–31]

Recent studies have shown the advantages of TCV from linking Vi-PS with other proteins, such as *Pseudomonas aeruginosa* recombinant exoprotein A (Vi-rEPA), a non-toxic mutant of diphtheria toxin (CRM197) protein (Vi-CRM_197_), and tetanus toxoid (TT) conjugate vaccine (Typbar-TCV and PedaTyph).[21,24,31,32–34] The DT has been proven to be safe and successfully used as carrier protein for meningococcal conjugate vaccine as the other carrier protein; CRM197 and TT.[33]

An animal study comparing Vi-DT and Vi-rEPA showed that the two vaccines elicited similar levels of anti-Vi IgG in mice.[24] The Vi-rEPA was safe and highly efficacious in 2–5 years old children in an endemic area.[13] Therefore, Vi-DT was predicted to be as immunogenic and protective as Vi-rEPA.[24]

Our study is a phase I clinical trial of a novel Vi-DT vaccine that was developed by conjugating the typhoid Vi-PS with the diphtheria toxoid. The phase I study using similar vaccine which was first developed by IVI had been conducted a year earlier in Filipino adults and children in three age groups; 18–45, 6–17 and 2–5 years.[25] We conducted our study in both adults and children groups in an age de-escalation manner (18–40 and 2–5 years) and continued following up all subjects until 6 months post vaccination. After V3 of the adults group, we continued the study in children. This vaccine aims to cover the limited availability of present TCV, particularly for younger children and infants in endemic countries. This vaccine has demonstrated to produce higher levels of anti-Vi IgG and to induce a booster response upon reinjection.[24] Therefore, in our study, we used two doses of the Vi-DT vaccine administered four weeks apart.

Our result revealed that Vi-DT and Vi-PS vaccines showed no difference in intensity of any immediate local and systemic adverse events within 30 minutes post-vaccination (Table 4). Overall, pain was the most common local reaction in the first 72 hours for both the Vi-DT and Vi-PS vaccines (Table 5). This is similar with results of phase I study in the Philippines that showed the pain injection site was the most common immediate reaction both in Test and Comparator vaccine groups.[25] Our study showed that muscle pain was the most common systemic reaction in the first 72 hours and generally occurred in the adult comparator group. The local and systemic AEs were primarily of mild intensities.

We compared the AEs of Vi-DT with Vi-PS in the two age groups after the first and second vaccination. In the adult group, majority subjects in Vi-DT group showed lower AEs than Vi-PS vaccine, but only any delayed systemic event within 30 minutes-24 hours revealed significant difference (Table 3). However, based on the causality assessment, all delayed AEs were unrelated to the vaccination. In the six-month follow up period, no other adverse events were reported, except two SAEs which occurred in one child subject. None of SAEs was deemed related to vaccine administration.

Regarding immunogenicity, the first and second doses of the typhoid Vi-DT conjugate vaccine were able to induce a seroconversion in all subjects. By contrast, only 84% to 92% subjects of Vi-PS group showed increased antibody titers 4-fold higher anti-Vi IgG (Table 6). This result has the same outcome with previous study which showed that Vi-DT vaccine induced immune response in all subjects where comparator vaccine only resulted in 97% subjects.[25]

In our study, 4 weeks after the first vaccination in both adults and children, the GMT were significantly higher after receiving the Vi-DT vaccine than those got Vi-PS vaccine (Table 7). However, in both groups, the second dose of Vi-DT did not induce a booster response. Moreover, there was a decrease in the GMT a month after the second dose. Study in the Philippine showed similar result, no further increase of GMT was detected post second dose of Vi-DT compared to post first dose.[25] These result was concomitant to other conjugate vaccines study. Antibody response was significantly higher in Vi-CRM_197_ group than Vi-PS group after the first vaccination but did not boost antibody titers after the second dose vaccination. Moreover, the second dose showed a reduction in antibody titers six months after second immunization.[11] Another study about *S*. *typhi* conjugate vaccine with tetanus toxoid protein (PedaTyph) also showed similar result.[31] By contrast, previous study using Vi-rEPA TCV revealed that there was a significant increase in IgG Vi four weeks after the second vaccination.[13]

Our findings showed that the GMT increased 95-fold in children and 86-fold in adults in Vi-DT group compared to their baseline levels (Table 7). Even though the result was similar to PedaTyph vaccine study, the final seroconversion revealed the different result. The PedaTyph vaccine did not show any seroconversion.[31]

Our study has some limitations. Our subjects were recruited from one study area, and the population was primarily composed of lower- to middle-income families. Thus, we cannot make comparison with other families with different income levels. Studies with a larger sample size should be conducted following our current study, involving children below two years old.

## Conclusions

Our phase I study suggests that the Vi-DT vaccine is safe and immunogenic in adults and children older than two years. A single dose of the vaccine is able to produce seroconversion and high GMT in all individuals.

## Supporting information

S1 File Protocol study typhoid 0116. (PDF)

S2 File CONSORT 2010 checklist. (PDF)

S3 File AJE certificate. (PDF)
