# Supplementary material for: Six-month follow up of a randomized clinical trial-phase I study in Indonesian adults and children: Safety and immunogenicity of Salmonella typhi polysaccharide-diphtheria toxoid (Vi-DT) conjugate vaccine
Source: PLoS One. 2019 Feb 13;14(2):e0211784. doi: 10.1371/journal.pone.0211784 (PMC6373931; doi:10.1371/journal.pone.0211784)
Supplement: S1 File — (PDF) [file pone.0211784.s001.pdf]

Safety and Immunogenicity of Vi-DT Typhoid Conjugate Vaccine (Bio Farma) in Adults and Children (Phase I)

Typhoid 0116
STUDY PROTOCOL

**Sponsor**	**PT BIO FARMA (PERSERO)**<br>Jl. Pasteur no.28 Bandung – 40161 INDONESIA
**Investigational Product**	Vi-DT Typhoid Conjugate Vaccine (Bio Farma)
**Manufacturing Sites**	PT Bio Farma, Jl. Pasteur no. 28 Bandung – 40161 Indonesia.
**Principal Investigator**	Bernie Endyarni Medise, dr, SpA(K), MPH.
**Medical Advisor**	Prof. Dr. Sri Rezeki Hadinegoro, dr., SpA(K).
**Sub investigators**	Prof. Dr. Soedjatmiko dr., SpA(K), M.Si<br>Dr. Hartono Gunardi,dr., SpA(K).<br>Dr. Hindra Irawan Satari, dr, SpA(K), M.TroPaed.<br>Dr. Iris Rengganis, dr., SpPD-KAI, FINASIM
**Field Investigators**	Prof. Dr. Rini Sekartini, dr., SpA(K).<br>Dr. Sukamto, dr., SpPD-KAI
**Biometry**	Dr. Aria Kekalih, dr., M.T.I
**Biological Laboratory**	Rini Mulia Sari, dr.<br>Lestari.
**Monitor**	Dr. Novilia Sjafri Bachtiar, dr., M.Kes.<br>Rini Mulia Sari, dr.<br>Mita Puspita, dr.<br>Asep Irham F. Q., dr.
**Date**	Desember 2016
**Version**	1.b

The information contained in this document is the property of Bio Farma and is confidential. It may be submitted to a Regulatory Authority, an Institutional Review Board/Ethics Committee, or an Investigator or a Pharmacist for the purpose of assessment in relation to registration of the product or initiation of a clinical trial. Reproduction or disclosure of the information in whole or in part is forbidden without the written consent of Bio Farma. This document must be returned to Bio Farma upon request

Title of study:
Safety and Immunogenicity of Vi-DT Typhoid Conjugate Vaccine (Bio Farma) in Adults and Children (Phase I)

Official Title:
A Randomized, Observer Blinded, Comparative, Phase I Safety Study in Two Age De-escalating Cohorts to Assess the Safety and Immunogenicity of Vi-DT Typhoid Conjugate Vaccine (Bio Farma) in Adults and Children (Phase I)

Study center :
Cipto Mangunkusumo Hospital/Department of Child Health School of Medicine, University of Indonesia, Jakarta.

Planned Study period: January - December 2017

Primary Objectives:
To assess the safety of Vi-DT vaccine in adults and children.

Secondary Objectives:
- To describe the safety of this vaccine following first and second dose immunization.
- To assess preliminary information of immunogenicity following Vi-DT vaccine immunization.

Current Primary Outcome:
- Local reaction and systemic event after vaccination (time frame: 28 days)
- Percentage of subjects with at least one immediate reaction (local reaction or systemic event) after vaccination.

Current Secondary Outcome:
- Adverse events after vaccination [Time Frame: 28 days]
- Percentage of subjects with at least one of these adverse events, solicited or not, within 24 h, 48h, 72h and 28 days after each vaccination.
- Serious adverse events after vaccination [Time Frame: 28 days]
- Number and percentage of subjects with serious adverse event from inclusion until 28 day after vaccination and up to 6 months after the last vaccination.
- Routine laboratory evaluation that probably related to the vaccination. [Time Frame: 7 days]
- Deviation from routine blood laboratory, kidney and liver function laboratory evaluation that probably related to the vaccination.
- Preliminary assessment of immunogenicity of typhoid conjugated vaccine (Vi-DT) [Time Frame: 28 days]
- Percentage of subjects with > 4 times increasing antibody
- Geometric Mean Titers (GMT) following immunization [Time Frame: 28 days]
- Geometric Mean Titers (GMT) 28 days following immunization

Study type: Interventional
Study phase: Phase 1

Study design:
Allocation: Randomized
Intervention Model: Parallel Assignment
Masking: Single (Investigator)
Primary Purpose: Prevention

Methodology:
Observer blinded, comparative, phase I safety study in two age de-escalating cohorts.

Condition: Safety Issues

Intervention:
Biological: Vi-DT (Bio Farma): Typhoid Conjugate Vaccine
Biological: one or two doses of Vi polysaccharide vaccine
Biological: 1 dose of Influenzae vaccine
Biological: 1 dose of Pneumococcal conjugate vaccine

Study Population:

- Healthy adults 18 - 40 years of age (study and comparator arms)
- Healthy children 2-5 years of age (study and comparator arms).

Sample size: 100 subjects @25 subjects per arm

Study Arms:
4 arms, 2 study and 2 comparator arms (Vi Polysaccharide):

1. Experimental: Vi-DT (Bio Farma)
   2 doses of 0.5 ml of Vi-DT Conjugated typhoid vaccine
   Intervention: Biological: Vi-DT (Bio Farma)

2. Active Comparator: Vi polysaccharide vaccine
   1 dose of 0.5 ml Vi polysaccharide vaccine + 1 dose of Influenzae Vaccine
   Interventions:
   Biological: Vi polysaccharide vaccine
   Biological: Influenzae vaccine

3. Experimental: Vi-DT (Bio Farma) ~ Children
   2 doses of 0.5 ml of Vi-DT Conjugated typhoid vaccine
   Intervention: Biological: Vi-DT (Bio Farma)

4. Active Comparator: Vi polysaccharide vaccine ~ Children
   1 dose of 0.5 ml Vi polysaccharide vaccine + 1 dose of Pneumococcal Conjugate Vaccine
   Interventions:
   Biological: Vi polysaccharide vaccine
   Biological: Pneumococcal conjugate vaccine

Eligibility
<u>Inclusion Criteria:</u>

1. Healthy
2. Subjects/Parents have been informed properly regarding the study and signed the informed consent form
3. Subject/Parents will commit to comply with the instructions of the investigator and the schedule of the trial

Exclusion Criteria:

1. Subject concomitantly enrolled or scheduled to be enrolled in another trial
2. Evolving mild, moderate or severe illness, especially infectious diseases or fever (axillary temperature ^{3} 37.5°C)
3. Known history of allergy to any component of the vaccines
4. History of uncontrolled coagulopathy or blood disorders contraindicating intramuscular injection
5. Subject who has received in the previous 4 weeks a treatment likely to alter the immune response (intravenous immunoglobulins, blood-derived products or long term corticotherapy (> 2 weeks).
6. Any abnormality or chronic disease which according to the investigator might interfere with the assessment of the trial objectives
7. Pregnancy & lactation (Adults)
8. Individuals who have previously received any vaccines against typhoid fever.
9. Subjects already immunized with any vaccine within 4 weeks prior and expect to receive other vaccines within 60 days following the first dose.
10. Individuals who have a previously ascertained typhoid fever.
11. History of alcohol or substance abuse.
12. Subject planning to move from the study area before the end of study period.

Evaluation Criteria

Primary Evaluation Criteria
The main evaluation criteria are number and percentage of subjects with at least one immediate reaction (local reaction or systemic event) within 30 minutes after vaccination.

Secondary Evaluation Criteria
- Number and percentage of subjects with at least one of these adverse events, solicited or not, within 24 h, 48h, 72h and 28 days after each vaccination.
- Number and percentage of subjects with serious adverse event from inclusion until 28 day after vaccination and up to 6 months after the last vaccination.
- Any deviation from routine laboratory evaluation that probably related to the vaccination.
- Description of safety data between groups

Immunogenicity: Preliminary assessment of immunogenicity of typhoid conjugated vaccine (Vi-DT) with Vi Polysaccharide vaccine in each cohort using the following criteria:
- Number and percentage of subjects with $\geq$ 4 times increasing antibody
- Geometric Mean Titers (GMT) following immunization
